# Hemophagocytic lymphohistiocytosis associated with acute otitis media: A case report

**DOI:** 10.1097/MD.0000000000038616

**Published:** 2024-06-21

**Authors:** Da Hyun Chung, Kyu-Yup Lee, Ji-Yoon Kim, Da Jung Jung

**Affiliations:** a Department of Otorhinolaryngology-Head and Neck Surgery, Kyungpook National University School of Medicine, Daegu, Korea; b Department of Pediatrics, School of Medicine, Kyungpook National University, Daegu, Korea.

**Keywords:** acute otitis media, case report, children, fever, hemophagocytic lymphohistiocytosis

## Abstract

**Introduction:**

Hemophagocytic lymphohistiocytosis (HLH) is a potentially life-threatening syndrome for which early recognition and treatment are essential for improving outcomes. HLH is characterized by uncontrolled immune activation leading to fever, cytopenias, hepatosplenomegaly, coagulation abnormalities, and elevated typical markers. This condition can be genetic or secondary, with the latter often triggered by infections. Here, we present a unique case of HLH secondary to acute otitis media (AOM), a common ear infection.

**Patient concerns:**

We describe a 4-year-old boy who initially presented with a high fever and otalgia, later diagnosed with bilateral AOM. Despite antibiotic treatment, his condition deteriorated.

**Diagnosis:**

The patient fulfilled diagnostic criteria for HLH.

**Interventions:**

Aggressive treatment by using combination therapy with immunoglobulins, intravenous steroids (dexamethasone), cyclosporine, and etoposide was performed.

**Outcomes:**

After 1 month of treatment, improvement in the otologic symptoms was observed, and hematological findings gradually improved and normalized.

**Lessions:**

The link between AOM and HLH may be associated with inflammatory responses and immunological mechanisms, highlighting the importance of considering HLH in severe infection cases. This case emphasizes the need for prompt diagnosis and management, especially in secondary HLH scenarios, to improve patient outcomes. It is imperative to be aware of the potential correlation between these 2 conditions, and healthcare professionals should consider the likelihood of HLH.

## 1. Introduction

Hemophagocytic lymphohistiocytosis (HLH) is a medical condition characterized by fever, enlargement of the spleen, low blood cell counts, and activated macrophages engulfing blood cells.^[1]^ This syndrome is life-threatening and is distinguished by the prolonged activation of cytotoxic T lymphocytes and natural killer (NK) cells.^[2]^ The intensity of this immune response can vary, encompassing mild splenomegaly and cytopenia, commonly observed in routine viral infections, and often resolve spontaneously. However, it can also escalate to an uncontrollable and severe reaction marked by coagulation abnormalities, profound deficits in blood cell numbers, and acidosis, ultimately culminating in fatal outcomes.^[3]^ There are both genetic and acquired forms of HLH, with the latter being referred to as macrophage activation syndrome, viral-associated hemophagocytic syndrome, or infection-associated hemophagocytic syndrome, depending on the triggering agents.^[3]^

The precise mechanisms leading to the development of HLH remain unclear. It is known to be rapidly progressive and lethal that if left untreated, the condition may result in fatal consequences like severe sepsis, and it is consistently linked with elevated mortality rates across all age groups.^[4]^ HLH is relatively uncommon, with a prevalence of approximately 1 in 100,000 individuals,^[5]^ predominantly involving pediatric and adolescent populations. The incidence of secondary HLH varies based on the underlying conditions and the population under study. It is more frequent than primary HLH, arising in the context of other diseases. However, specific incidence rates can be difficult to determine due to the rarity of the syndrome and the wide range of associated conditions.^[6]^

The most common health issues in children involve respiratory infections affecting the upper airway, which includes ear infections (otitis media).^[7]^ The term “otitis media” encompasses various conditions related to middle ear symptoms, including the acute or chronic suppurative types or those with effusions. Persistent symptoms and treatment failures of otitis media can lead to complications that can damage structures of the middle ear, such as retraction pockets, adhesions, perforations, ossicular erosion, and cholesteatoma, as well as other intratemporal and intracranial problems.^[8]^

Although infection-triggered HLH, a condition characterized by excessive and pathologic inflammation, can affect patients with various medical conditions, there has been limited research on the instances of HLH in individuals initially diagnosed with acute otitis media (AOM). We present a case of a 4-year-old male child with HLH secondary to AOM.

## 2. Case report

A previously healthy 4-year-old boy presented with a sudden high fever and sore throat, initially treated with cefotaxime at a local medical center. As the fever continued, he complained of otalgia, leading to the replacement of ceftriaxone with netilmicin after 3 days. Despite receiving empirical antibiotics and symptomatic treatment for an unknown infection, his symptoms did not improve.

**Figure 1.**
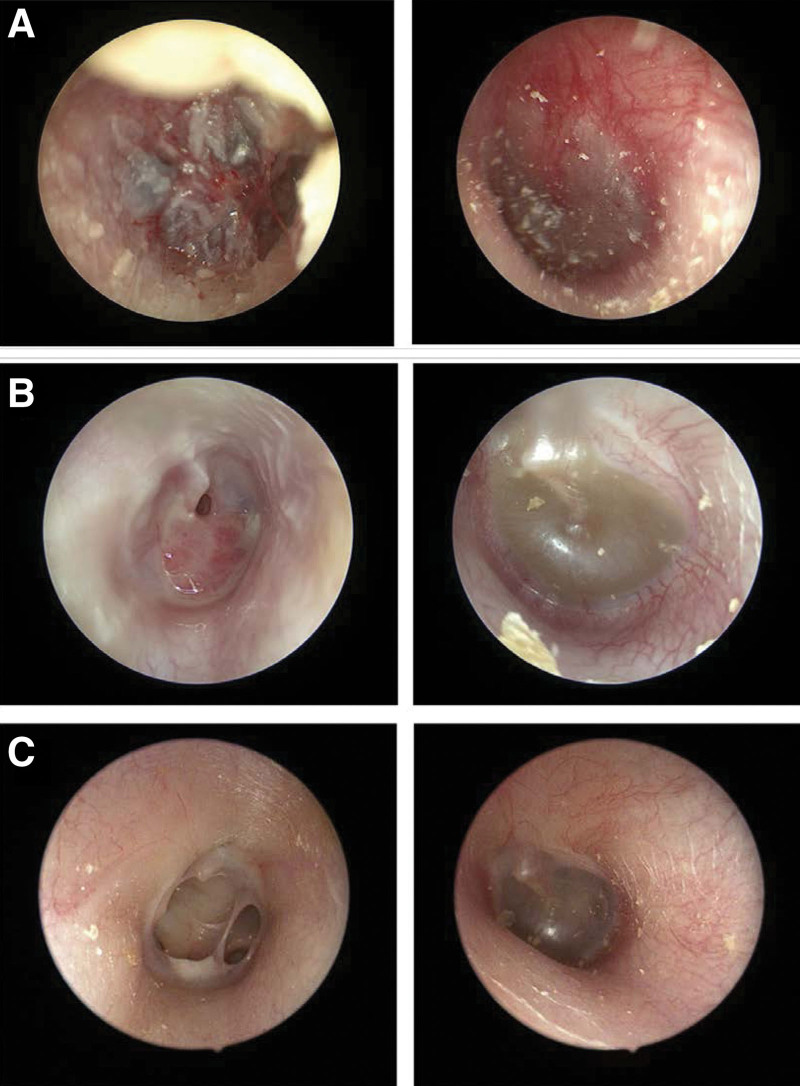
(A) Acute hemorrhagic otitis media in right ear. White colored epithelium is seen on the tympanic membrane with small perforation. Hyperemia is seen in left ear. (B) Suppurative stage in acute otitis media is seen in right ear and small perforation in tympanic membrane is seen after cleaning the purulent material. Otitis media with effusion and amber colored fluid is seen in left ear. (C) Recent otoscopic findings showing tympanic membrane perforation in right ear, but no sign of inflammation or recurrence.

Upon admission to our hospital, blood tests revealed leukocytosis (WBC 17,030/µL) and thrombocytosis (Platelet 757 K/µL), elevated ESR (74 mm/h), and C-reactive protein (CRP) (5.1 mg/dL). Respiratory PCR tests were positive for adenovirus and *Mycoplasma pneumoniae*, with Mycoplasma IgM titer > 27, indicating a positive result. The patient continued to complain of otalgia and otorrhea, leading to a consultation with our department. Given the diagnosis of bilateral AOM, antibiotics were continued, and myringotomy with tube insertion was to be considered, with close monitoring for improvement (Fig. 1A). After 3 days of hospitalization, the fever subsided, and he was discharged with oral Amoxicillin/Clavulanate.

**Figure 2.**
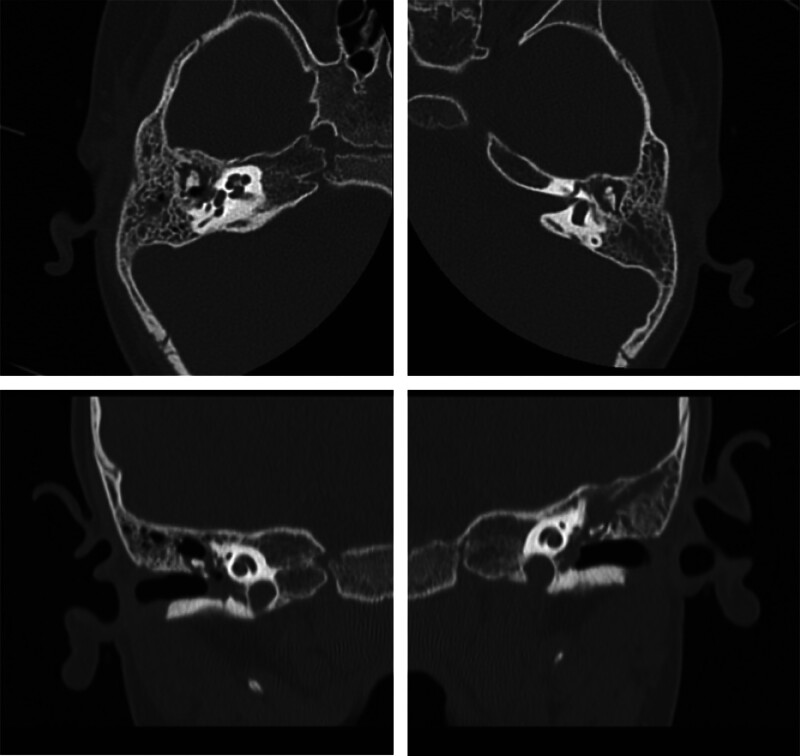
Near total opacification in bilateral middle ear cavity and mastoid air cells. No evidence of ossicle or bone erosions.

During follow-up outpatient visits, right otitis media and left ear effusion persisted despite the use of Sulfamethoxazole/Trimethoprim based on bacterial culture results, which revealed *Staphylococcus epidermidis* (Fig. 1B). Continued oral antibiotics and ear dressing improved the condition slightly, but the ear infection persisted. A repeat bacterial culture identified *Acinetobacter baumannii*, and a temporal computer tomography (CT) scan showed evidence of bilateral mastoiditis (Fig. 2), leading to admission to our department for intravenous antibiotics and V-tube insertion.

**Figure 3.**
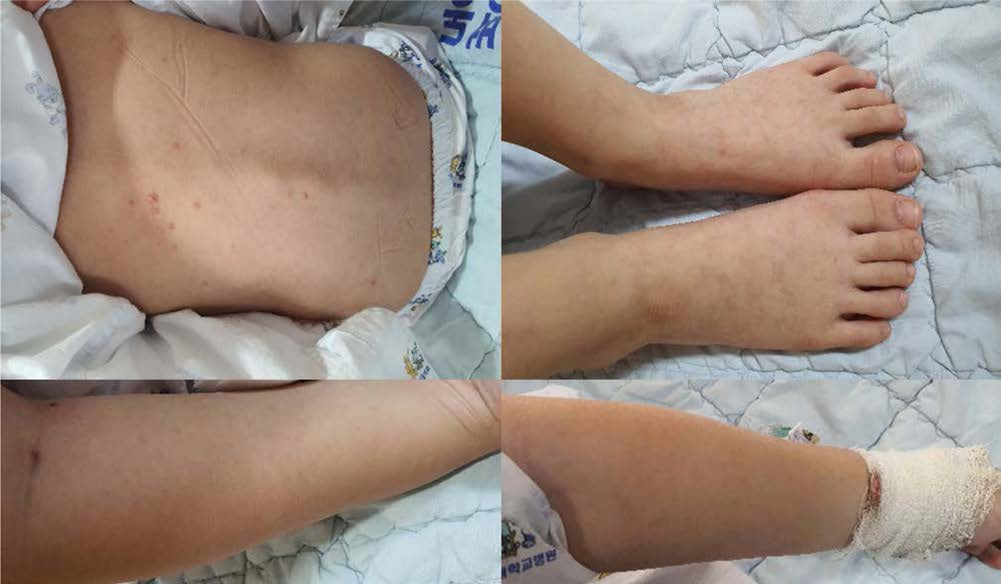
Development of whole-body skin rash with abnormal laboratory findings.

Blood tests during this hospitalization revealed no specific abnormalities (WBC 9220/µL, CRP < 0.1 mg/dL, and normal liver function). Piperacillin–Tazobactam (250 mg/kg/day divided into 3 doses) was initiated in collaboration with the pediatric department, and left V-tube insertion was performed under general anesthesia. After 2 weeks of hospital treatment, otorrhea improved, and discharge planning was being considered. However, just before discharge, he developed a fever and a skin rash all over the body (Fig. 3), prompting further blood tests. The results showed bicytopenia (ANC 0.5 K/μL, PLT 81 K/μL), abnormal liver function (aspartate aminotransferase (AST)/alanine aminotransferase (ALT) 1595/945 U/L, lactate dehydrogenase (LDH) 5328 U/L), elevated Ferritin (72,456 ng/mL), CRP (7.64 mg/dL), and prolonged PT/aPTT/INR (50%/45″/1.5).

**Figure 4.**
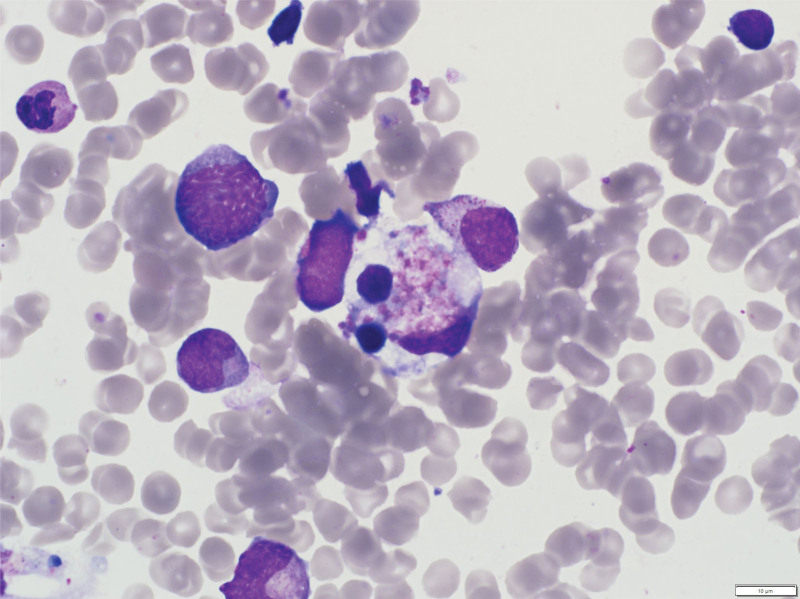
Photomicrograph of a bone marrow smear from a patient with hemophagocytic lymphohistiocytosis, showing foamy macrophages engulfing mature and precursor erythrocytes.

To investigate the cause of the persistent fever, leukopenia, and elevated ferritin, a bone marrow examination was conducted to screen for HLH. The bone marrow biopsy showed a cellularity of 80%, and the bone marrow aspirate revealed evidence of hemophagocytosis by histiocytes, confirming the diagnosis of HLH (Fig. 4). The patient was started on combination therapy with immunoglobulins, intravenous steroids (dexamethasone), cyclosporine, and etoposide as part of the complex chemotherapy regimen.

**Figure 5.**
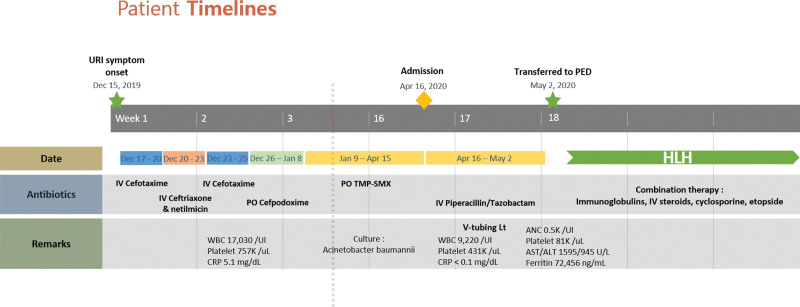
Timeline of antibiotics, laboratory results, and overall treatments according to day of illness and day of hospitalization.

After 1 month of treatment, improvement in the otologic symptoms was observed, and hematological findings gradually improved and normalized. The patient continued to be monitored as an outpatient for 9 months, undergoing combination chemotherapy without evidence of recurrence. HLH treatment has been terminated and is under observation without any signs of recurrence (Fig. 1C). Figure 5 shows a timeline of antibiotics, laboratory results, and overall treatments for a patient according to the day of illness and day of hospitalization. The patient’s legal guardian provided informed consent for the publication of this case report details and images.

## 3. Discussion

HLH is a syndrome characterized by persistent fever, cytopenia, hepatosplenomegaly, coagulation abnormalities, and elevated levels of specific biomarkers such as ferritin and soluble interleukin-2 (IL-2) receptor (sIL-2R).^[2]^ The diagnostic criteria for HLH are based on guidelines proposed in 1991 and revised in 2004.^[9]^ To confirm a diagnosis of HLH, at least 5 out of the following 8 criteria must be present (Table 1).^[9]^ Additionally, patients may develop symptoms such as skin rashes, hepatitis, disseminated intravascular coagulation, acute liver failure, central nervous system involvement, multiorgan failure, and various other complications, often resulting in a high mortality rate. Therefore, the timely recognition and treatment of this hyperinflammatory syndrome are crucial.^[2]^ HLH is categorized into 2 main types: primary HLH, which has a genetic basis, and secondary HLH, which is an acquired condition primarily associated with infections, malignancies, or autoimmune disorders. In the case of our patient, he fulfilled 5 of the 8 diagnostic criteria for HLH. Following the exclusion of malignancies and autoimmune diseases as potential causes, our attention turned to identifying treatable infectious factors that might be responsible for triggering HLH.

**Table 1:** Diagnostic criteria for HLH used I the HLH-2004 trial.

The diagnosis of HLH may be established:
A. Molecular diagnosis consistent with HLH: pathologic mutations of PRF1, UNC13D, Munc18-2, Rab27a, STX11, SH2D1A, or BIRC4
B. Five of the 8 criteria listed below are fulfilled:
1. Fever ≥ 38.5 °C
2. Splenomegaly
3. Cytopenias (affecting at least 2 of 3 lineages in the peripheral blood)
Hemoglobin < 9 g/dL (in infants < 4 wk: hemoglobin < 10 g/dL)
Platelets < 100 × 10^3^/mL
Neutrophils < 1 × 10^3^/mL
4. Hypertriglyceridemia (fasting > 265 mg/dL) and/or hypofibrinogenemia (<150 mg/dL)
5. Hemophagocytosis in bone marrow, spleen, lymph nodes, or liver
6. Low or absent NK-cell activity
7. Ferritin > 500 ng/mL
8. Elevated sCD25 (α-chain of sIL-2 receptor)

HLH = hemophagocytic lymphohistiocytosis, IL = interleukin, NK = natural killer.

AOM and HLH have a generally uncommon direct connection. However, in cases where AOM progresses to severe complications, consideration of immunological mechanisms and the possibility of HLH may be warranted.

AOM typically starts as ear inflammation, but in severe cases, the infection and inflammation can extend beyond the ear, involving the immune system. Pathophysiology of HLH can be described as a hyperinflammatory syndrome with an uncontrolled immune response. The pathogenesis of HLH has recently been elucidated, which includes impaired lymphocyte activation in response to immune stimulation and interference of cytokine balances.^[10]^ This malfunction leads to the excessive production of inflammatory cytokines, which subsequently drive the infiltration of macrophages and the formation of a complex cytokine network.^[10]^

The pathogenesis of AOM is mainly due to the inflammatory response to the preceding infections, which includes change of vascular permeability, chemotaxis of cytotoxic T lymphocytes and B lymphocytes, and synthesis of cytokines. Such responses can occasionally cause impaired lymphocyte activation and interference of cytokine balances, which may lead to excessive immune activation and possibly trigger the development of HLH. Consequently, in cases where AOM co-occurs with other infections or immune-related issues, it is essential to consider the possibility of HLH as part of the diagnostic process.

HLH that occurs in patients without a genetic predisposition to HLH owing to a significant immunological trigger is referred to as secondary HLH. It can develop in association with various infections, including deoxyribonucleic acid (DNA) viruses (e.g., Epstein–Barr virus [EBV], cytomegalovirus, parvovirus, herpes simplex virus, and adenovirus) and intracellular pathogens (e.g., Leishmania).^[11]^ Recent analyses shed light on the mechanisms underlying the relationship between viral infections and HLH. A research suggests that certain viruses, particularly EBV, can directly infect and activate immune cells, leading to an exaggerated immune response.^[12]^ Such dysregulated immune activation can result in the hallmark features of HLH, such as fever, cytopenias, hepatosplenomegaly, and hyperinflammation. Moreover, in association with genetic predispositions, mutations in genes associated with immune regulation, such as those involved in the perforin–granzyme pathway or the cytokine signaling pathway, can increase susceptibility to HLH following viral exposure.^[12,13]^ Several studies have reported cases of patients with severe COVID-19 exhibiting features consistent with HLH due to immunological hyperactivation.^[14]^ However, the range of infections linked to HLH is extensive and can vary by geographic region, season, and socioeconomic status.^[11]^ Furthermore, secondary HLH can occur in the context of other underlying conditions, including autoimmune disorders such as inflammatory bowel disease(IBD).^[15]^ In patients with IBD, HLH may develop as a complication of the disease itself or as a side effect of immunosuppressive therapies used to manage IBD flare-ups.^[15]^

Given the underlying mechanism involving an excess of inflammatory mediators in HLH, the primary objective of treatment is to attenuate circulating cytokines and provide support for organ dysfunction. Currently, HLH treatment adheres to the HLH-1994 and HLH-2004 protocols, encompassing immunosuppressive regimens consisting of dexamethasone, cyclosporine, and etoposide.^[9]^ Dexamethasone, a potent corticosteroid, plays a pivotal role in mitigating the immune response, particularly its inflammatory component. Cyclosporine A is employed to inhibit lymphocyte activity and interfere with macrophage function. Etoposide is especially important in severe cases of HLH, as it reduces activated T-cells, a key player in the immune response.

In cases where HLH is triggered by an infection, the treatment approach may encompass high-dose intravenous immunoglobulins (IVIG) and antimicrobial agents. The combination of IVIG with steroids can be advantageous in infection-induced HLH, aiding in the modulation of the immune response. Moreover, timely initiation of antimicrobial agents upon presentation is imperative to address the underlying infection that may have precipitated HLH.

A substantial proportion of patients receiving this combination of treatments exhibit partial responses and improvements in their laboratory test results. Additionally, it is essential for all HLH patients to receive comprehensive supportive care to manage organ dysfunction and complications associated with the condition. This comprehensive approach aims to control the excessive immune response, address the root cause, and promote the overall well-being of the patient. Prognosis for individuals with secondary HLH can vary widely depending on several factors, including the underlying cause, the promptness of diagnosis, and the effectiveness of treatment.

The prognosis of HLH varies, and it can sometimes lead to severe, even fatal, outcomes. Of course, there have been no reports of progression from otitis media to HLH as in this case, and such occurrences are extremely rare. While there are limitations in identifying the specific causative agents that trigger HLH directly, both conditions share a common foundation involving inflammatory responses associated with infection and immunological mechanisms.

HLH can be challenging to diagnose because of its atypical symptoms, diagnostic limitations, and diverse triggering factors, making a specific cause difficult to pinpoint. In the present case, it was initially difficult to associate AOM with HLH. However, abnormal blood test results and symptoms which differed from those of simple AOM, such as a skin rash, raised suspicion and led to the diagnosis.

To date, no case reports have been published on the association between AOM and HLH. However, recent studies have indicated a correlation between common illnesses and severe HLH. This finding could contribute to the expansion of medical knowledge and offer a new perspective. This underscores the importance of considering the potential links between AOM or other common illnesses and HLH, emphasizing the need for early HLH diagnosis and effective treatment. Such findings are expected to aid healthcare practitioners in approaching patient care with fresh insights into real clinical settings.

In conclusion, it is important not to overlook the potential association between these 2 conditions. In situations involving severe infections, healthcare professionals must remain vigilant, considering the possibility of HLH, and may need to seek guidance from infectious disease specialists to ensure prompt diagnosis and effective management.

## Author contributions

**Conceptualization:** Da Hyun Chung, Kyu-Yup Lee, Da Jung Jung.

**Data curation:** Da Hyun Chung.

**Writing – original draft:** Da Hyun Chung.

**Writing – review & editing:** Da Hyun Chung, Kyu-Yup Lee, Da Jung Jung.

**Investigation:** Ji-Yoon Kim.

**Project administration:** Da Jung Jung.
